# Neutralization of Bacterial YoeB_Spn_ Toxicity and Enhanced Plant Growth in *Arabidopsis thaliana* via Co-Expression of the Toxin-Antitoxin Genes

**DOI:** 10.3390/ijms17040321

**Published:** 2016-04-20

**Authors:** Fauziah Abu Bakar, Chew Chieng Yeo, Jennifer Ann Harikrishna

**Affiliations:** 1 Centre for Research in Biotechnology for Agriculture (CEBAR) and Institute of Biological Sciences, Faculty of Science, University of Malaya, 50603 Kuala Lumpur, Malaysia; afung87@gmail.com; 2 Biomedical Research Centre, Faculty of Medicine, Universiti Sultan Zainal Abidin, Medical Campus, 21300 Kuala Terengganu, Malaysia; chewchieng@gmail.com

**Keywords:** bacterial toxin-antitoxin, YefM antitoxin, YoeB toxin, 17-β-estradiol induction, heterologous expression, *Arabidopsis thaliana*

## Abstract

Bacterial toxin-antitoxin (TA) systems have various cellular functions, including as part of the general stress response. The genome of the Gram-positive human pathogen *Streptococcus pneumoniae* harbors several putative TA systems, including *yefM-yoeB_Spn_*, which is one of four systems that had been demonstrated to be biologically functional. Overexpression of the *yoeB_Spn_* toxin gene resulted in cell stasis and eventually cell death in its native host, as well as in *Escherichia coli*. Our previous work showed that induced expression of a *yoeB_Spn_* toxin-Green Fluorescent Protein (*GFP*) fusion gene apparently triggered apoptosis and was lethal in the model plant, *Arabidopsis thaliana*. In this study, we investigated the effects of co-expression of the *yefM_Spn_* antitoxin and *yoeB_Spn_* toxin-*GFP* fusion in transgenic *A. thaliana*. When co-expressed in Arabidopsis, the YefM_Spn_ antitoxin was found to neutralize the toxicity of YoeB_Spn_-GFP. Interestingly, the inducible expression of both *yefM_Spn_* antitoxin and *yoeB_Spn_* toxin-*GFP* fusion in transgenic hybrid Arabidopsis resulted in larger rosette leaves and taller plants with a higher number of inflorescence stems and increased silique production. To our knowledge, this is the first demonstration of a prokaryotic antitoxin neutralizing its cognate toxin in plant cells.

## 1. Introduction

Toxin-antitoxin (TA) systems are extensively found in bacteria and archaea, where they play diverse roles in important cellular functions. Bacterial toxin-antitoxin (TA) systems usually consist of a pair of genes encoding a stable toxin and its cognate labile antitoxin and are located in the chromosome or in plasmids of most bacterial species [1,2]. Currently, TA systems have been classified into five types (types I–V) according to how the antitoxins counteract the effect of their cognate toxins [2,3,4,5,6]. However, the most studied TA systems are of type II, in which the protein antitoxin inhibits the toxin protein through tight binding, with the antitoxin blocking the toxin active site [1,7].

Most of the characterized TA system toxins function as endoribonucleases, while other toxins disrupt DNA replication and transcription. Some toxins may also interfere with the synthesis of the bacterial cell wall [7,8]. Among these bacterial toxins, some have been shown to be functional when expressed in eukaryotic cells. They have the potential for use in the manipulation of eukaryotic cell growth, such as in restricting the accidental escape of genetically-modified cells [9]. The bacterial RelE toxin from *E. coli* has been shown to be functional in the yeast *Saccharomyces cerevisiae*, where induction of the toxin gene in transgenic yeast cells inhibited their growth. When expressed in human osteosarcoma cells, RelE was also shown to trigger apoptosis [10].

The Gram-positive bacterium, *Streptococcus pneumoniae* (the pneumococcus), is a common cause of human respiratory tract infections and has been associated with outstanding morbidity and mortality [11]. Up to 10 putative type II pneumococcal TA systems have been identified [12]. Of these, four have been demonstrated to be functional, namely *relBE2*, *yefM-yoeB_Spn_*, *pezAT* and *phd-doc* [13,14]. The *yefM-yoeB_Spn_* TA system has been shown to be functional with overexpression of the *yoeB_Spn_* toxin, leading to cell stasis and eventually cell death in both *S. pneumoniae* and *E. coli* [15]. Our previous work [16] produced transgenic *Arabidopsis thaliana* carrying a *yoeB_Spn_* chromosomal toxin-Green Fluorescent Protein (*GFP*) fusion gene expressed using a 17-β-estradiol inducible two-component expression system. We showed that expression of the *yoeB_Spn_* toxin-*GFP* fusion gene apparently triggered apoptosis, which resulted in lethality in *A. thaliana*; therefore, we suggested that the conditional expression of *yoeB_Spn_* toxin could be used to ablate pollen formation for the development of male sterile plants for containment of transgenic plants or for hybrid seed production [16]. In the current study, we investigated the effects of co-expressing the *yefM*_Spn_ antitoxin and *yoeB_Spn_* toxin in *A. thaliana* by cross-pollination between plants carrying either the *yefM_Spn_* antitoxin or *yoeB_Spn_* toxin-*GFP* fusion in inducible plant gene expression constructs.

## 2. Results

### 2.1. Production of yefM_Spn_ Antitoxin in Transgenic A. thaliana

**Figure 1:**
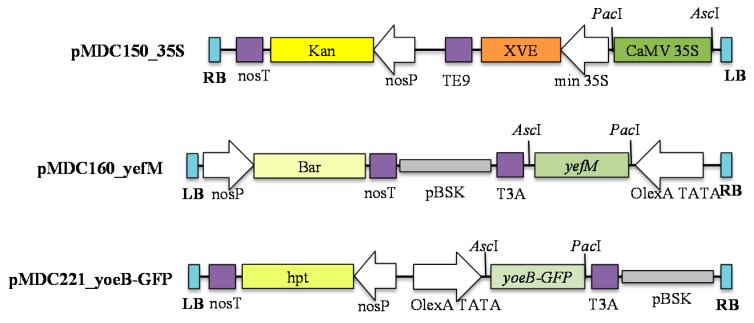
Map of the recombinant constructs used in this study. The T-DNA region of the recombinant activator vector pMDC150_35S and the recombinant responder vectors pMDC160_yefM and pMDC221_yoeBGFP are shown. The left border (LB) and right border (RB) regions flanking the T-DNA are indicated in blue boxes. The transgenes of interest (the cauliflower mosaic virus (CaMV) 35S promoter in pMDC150_35S, *yefM_Spn_* in pMDC160_yefM and the *yoeB_Spn_-GFP* fusion in pMDC221_yoeB-GFP) were cloned between the *Asc*I and *Pac*I unique restriction sites via Gateway recombination. In pMDC150_35S, expression of the XVE activator is driven by the CaMV 35S promoter and is, thus, constitutively expressed. Expression of the transgenes *yefM_Spn_* (in pMDC160_yefM) and *yoeB_Spn_-GFP* (in pMDC221_yoeB-GFP) are driven by the XVE-responsive promoter (labeled as *OlexA TATA*) and are, thus, inducible by 17-β-estradiol [16,17]. The plant selection markers (in yellow boxes and labeled as: Bar, Basta resistance gene; Kan, kanamycin resistance gene; hpt, hygromycin resistance gene) are driven by the *nos* promoter (labeled as *nosP*). TE9, T3A and *nosT* are transcriptional terminators (indicated in purple boxes); pBSK, pBlueScript backbone (indicated as a grey box).

**Figure 2:**
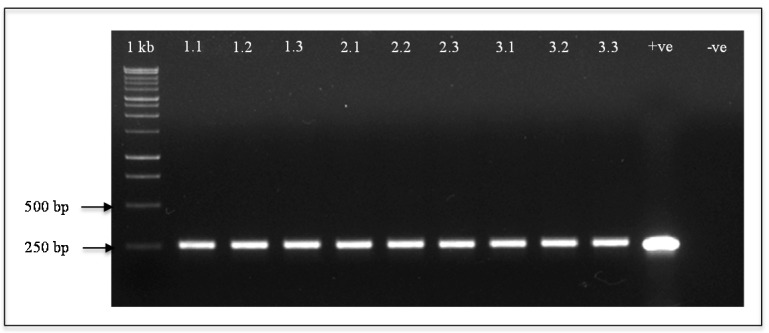
Detection of the *yefM_Spn_* antitoxin in transformed *Arabidopsis thaliana*. Extracted DNA from transformed plants of three different T_2_ lines was used as the template for PCR (Lanes **1.1**–**1.3**: transgenic Line 1; Lanes **2.1**–**2.3**: transgenic Line 2; Lanes **3.1**–**3.3**: transgenic Line 3) using *yefM_Spn_*-specific primers. Lane **+ve**: pMDC160_*yefM_Spn_* as the positive control. Lane **–ve**: wild-type *A. thaliana* Col 0 as the negative control. 1 kb: 1-kb DNA ladder (Fermentas). Molecular sizes are indicated.

In this study, a 17-β-estradiol-inducible two-component system [17] was used to produce transgenic *A. thaliana* for controlled expression of the *yefM_Spn_* antitoxin gene. The *yefM_Spn_* antitoxin gene was cloned in the responder vector pMDC160 (resulting in the recombinant designated pMDC160_yefM), while the cauliflower mosaic virus (CaMV) 35S promoter was cloned into the activator vector pMDC150 (as the recombinant pMDC150_35S [16]) to drive the constitutive expression of the 17-β-estradiol-responsive XVE transcriptional activator (Figure 1). These two constructs were introduced into *A. thaliana* by the floral dip method, and five independent transgenic lines (T_0_) were obtained by screening under kanamycin and Basta selection. After subsequent screening on the kanamycin-Basta mixture, three independent T_1_ transgenic lines were chosen for further analysis and used to produce 68 Basta- and kanamycin-resistant transgenic T_2_ lines. The introduction of the *yefM_Spn_* antitoxin into the plant genome was confirmed by PCR analysis of three randomly-selected *yefM_Spn_*-expressing plants from each T_2_ line (Lines 1, 2 and 3) (Figure 2, Lanes 1.1–3.3). All of the T_2_ progeny tested contained the expected 255-bp band corresponding to the *yefM_Spn_* DNA fragment, indicating that the transgene was successfully transferred into *A. thaliana*.

### 2.2. Transgenic A. thaliana Showed Normal Morphology after 17-β-Estradiol Induction for the Expression of yefM_Spn_ Antitoxin

**Figure 3:**
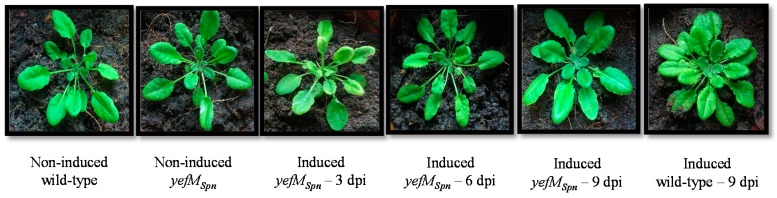
Four-week-old T_2_ transgenic *A. thaliana* plants harboring the *yefM_Spn_* transgene 3, 6 and 9 days post-induction (dpi) with 17-β-estradiol. Also depicted are non-induced wild-type *A. thaliana*, non-induced transgenic *A. thaliana* (*yefM_Spn_*) and induced wild-type *A. thaliana* applied with 17-β-estradiol at nine days post-induction.

The growth of the transgenic *A. thaliana* (*yefM_Spn_*) plants induced with 17-β-estradiol to express *yefM_Spn_* was not distinguishable from that of the control plants, *i.e.*, 17-β-estradiol-induced *A. thaliana* wild-type, non-induced wild-type and non-induced *A. thaliana* (*yefM_Spn_*) transgenic plants, with no morphological differences in rosette leaves and shape, even nine days after induction (Figure 3). Similar results were observed for all three independent lines.

### 2.3. Crosses of T_1_ Transgenic yoeB_Spn_-GFP Plants with T_1_ Transgenic yefM_Spn_ Plants Produced yefM_Spn_ × yoeB_Spn_-GFP Hybrid Lines

**Figure 4:**
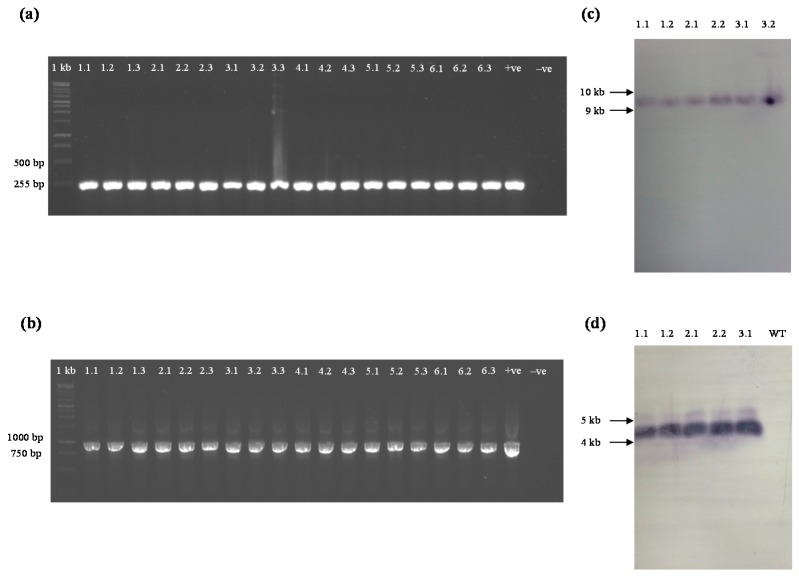
Detection of *yefM_Spn_* and *yoeB_Spn_-GFP* transgenes in hybrids of transgenic *A. thaliana* plants. (**a**) PCR detection using *yefM_Spn_*-specific primers (Lanes **1.1**–**1.3**: hybrid Line 1; Lanes **2.1**–**2.3**: hybrid Line 2; Lanes **3.1**–**3.3**: hybrid Line 3; Lanes **4.1**–**4.3**: hybrid Line 4; Lanes **5.1**–**5.3**: hybrid Line 5; Lanes **6.1**–**6.3**: hybrid Line 6) and showing the expected amplified product of 255 bp; lane **+ve**: pMDC160_yefM_Spn_; lane −ve: wild-type plant; 1 kb: 1-kb DNA ladder (Fermentas); (**b**) PCR detection of the hybrid Arabidopsis plants using *yoeB_Spn_-GFP*-specific primers (Lanes **1.1**–**1.3**: hybrid Line 1; Lanes **2.1**–**2.3**: hybrid Line 2; Lanes **3.1**–**3.3**: hybrid Line 3; Lanes **4.1**–**4.3**: hybrid Line 4; Lanes **5.1**–**5.3**: hybrid Line 5; Lanes **6.1**–**6.3**: hybrid Line 6) and showing the expected amplified product of 990 bp; lane **+ve**: pMDC221_*yoeB_Spn_-GFP*; lane **−ve**: wild-type plant; 1 kb: 1-kb DNA ladder (Fermentas); (**c**) Southern blotting analysis using *yefM_Spn_* as a probe (1.1, 1.2: hybrid Line 1; 2.1, 2.2: hybrid Line 2; 3.1, 3.2: hybrid Line 3); and (**d**) Southern blotting analysis using *GFP* as a probe on *Eco*RI-digested genomic DNA (1.1, 1.2: hybrid Line 1; 2.1, 2.2: hybrid Line 2; 3.1: hybrid Line 3, WT: non-transgenic plant).

All T_0_ transgenic *yoeB_Spn_-GFP* and *yefM_Spn_* plants were capable of self-pollination and produced normal seeds. The seeds were harvested and germinated under selection. The T_1_ *yoeB_Spn_-GFP* plants were crossed with T_1_ *yefM_Spn_* plants, and the seeds were harvested. A total of 237 hybrid plants from six different lines survived under antibiotic/herbicide selection. Three plants were chosen from each line for PCR to confirm the presence of *yefM_Spn_* (Figure 4a) and *yoeB_Spn_-GFP* (Figure 4b). Genomic DNA from these PCR-positive plants was examined by Southern blotting, and all tested transgenic hybrid plants showed the expected hybridization signals, indicating the likely integration of the *yefM_Spn_* (Figure 4c) and *yoeB_Spn_-GFP* (Figure 4d) transgenes into the genome of the respective transgenic hybrid lines.

### 2.4. Hybrids of Transgenic Plants Expressed Both yefM_Spn_ and yoeB_Spn_-GFP after Induction with 17-β-Estradiol

**Figure 5:**
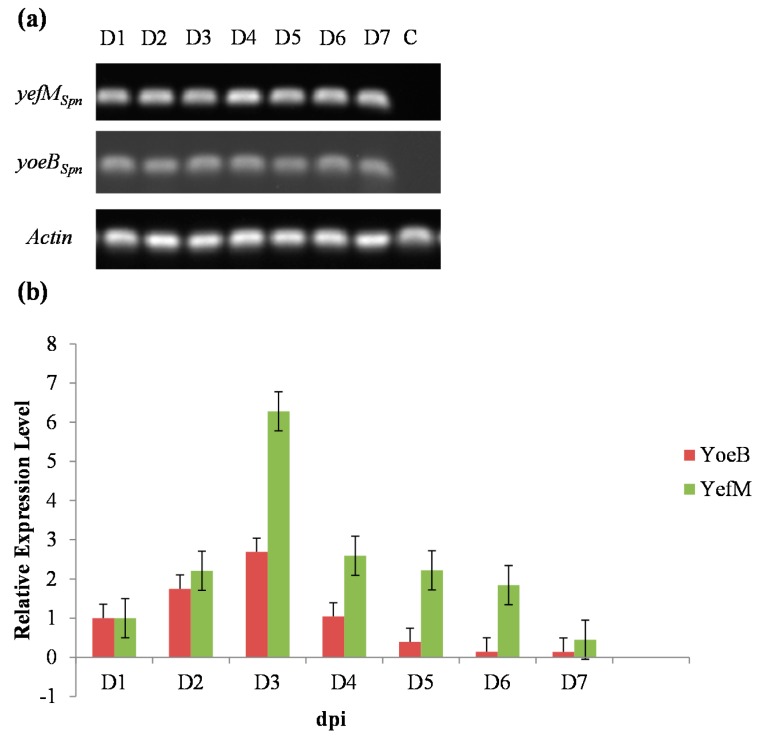
The relative expression levels of *yefM_Spn_* and *yoeB_Spn_-GFP* transcripts in hybrid plants as determined by qRT-PCR from Day 1 (D1)–Day 7 (D7) after 17-β-estradiol induction. (**a**) Agarose gel electrophoresis following RT-PCR to detect the transgene transcript in the hybrid *A. thaliana* plants harboring the *yefM_Spn_* and *yoeB_Spn_-GFP* transgenes. C, wild-type *A. thaliana* as the control; and (**b**) qRT-PCR analysis of *yefM_Spn_* and *yoeB_Spn_-GFP* in hybrid plants. Data were normalized to the endogenous *A. thaliana* actin gene (*Actin*), and the sample taken at Day 1 was set to 1.0. Error bars represent the standard deviation from the RT-PCR amplification of three biological replicates of *A. thaliana* rosette leaves’ total RNA samples.

Before induction, the *yefM_Spn_* × *yoeB_Spn_-GFP* hybrid plants grown in selective media did not show any signs of abnormality, and we detected no expression of either transgene by RT-PCR. The RT-PCR analysis with total RNA extracted from rosette leaf tissues after induction with 100 μM 17-β-estradiol confirmed the transcription of both genes from Days 1–7 after induction (Figure 5a). The relative expression levels of *yefM_Spn_* and *yoeB_Spn_-GFP* were analyzed by qRT-PCR using the rosette leaves from the same plants for Days 1–7 after induction (Figure 5b). The transcript levels of *yefM_Spn_* and *yoeB_Spn_-GFP* each increased over the first three days, after which they decreased with *yefM_Spn_* showing higher relative expression levels than *yoeB_Spn_-GFP* from Day 2 post-induction.

### 2.5. Induced Expression of yefM_Spn_ and yoeB_Spn_-GFP Enhanced Growth in Hybrid A. thaliana

**Figure 6:**
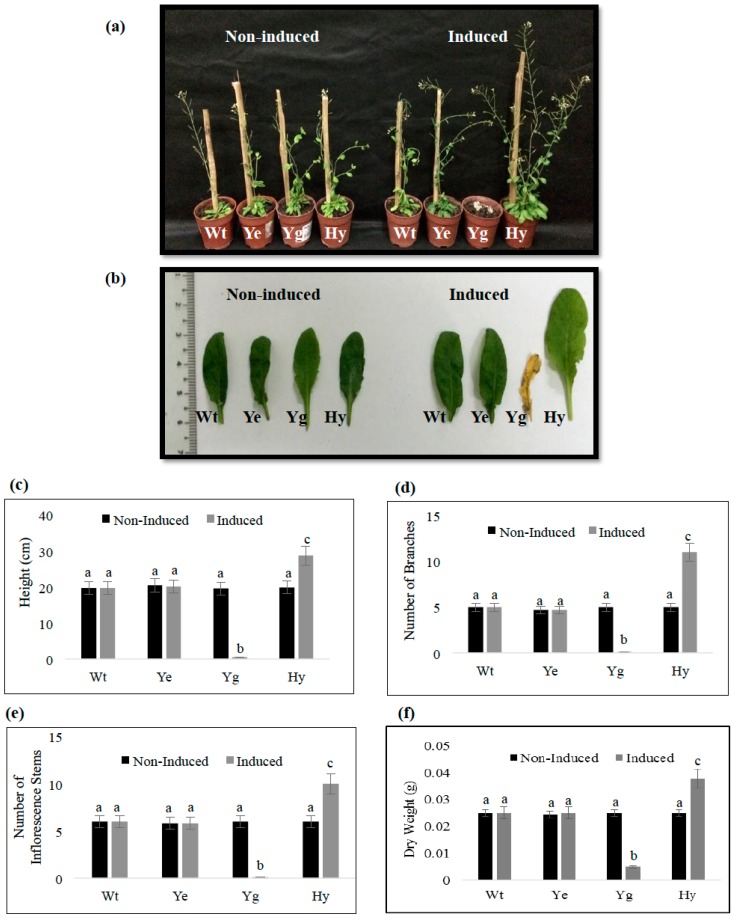
Morphology of the *yefM_Spn_* × *yoeB_Spn_-GFP* transgenic hybrid and control plants at nine weeks. (**a**) Non-induced and induced plants at nine weeks after planting. Plants were induced with 17-β-estradiol four weeks after planting and were allowed to grow until maturity; (**b**) The seventh leaf of the rosette of the hybrid plants (Hy) as compared to the wild-type (Wt), *A. thaliana* (*yefM_Spn_*) (Ye) and *A. thaliana* (*yoeB_Spn_-GFP*) (Yg); (**c**) Total height for each plant measured from the soil to the top of the plant; (**d**) Number of branches bearing siliques in each plant; (**e**) Number of inflorescence stems formed in each plant; and (**f**) Dry weight of each plant. Wt: wild-type *A. thaliana*; Ye: transgenic *A. thaliana* (*yefM_Spn_*); Yg: transgenic *A. thaliana* (*yoeB_Spn_-GFP*); Hy: transgenic *A. thaliana* (*yefM_Spn_* × *yoeB_Spn_-GFP*) hybrid. The data in (**c**–**f**) are shown as the mean ± standard deviation (*n* = 20). Different letters above the bars indicate significantly different means (*p* < 0.05, as analyzed by one-way ANOVA (Tukey used as the *post hoc* test)).

**Figure 7:**
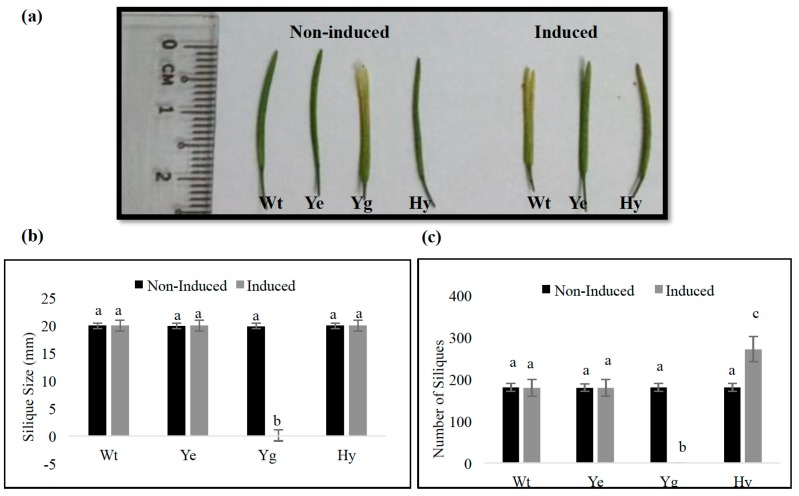
Silique phenotypes in induced and non-induced plants at nine weeks after germination. (**a**) Silique size of non-induced and induced plants; (**b**) Comparison of mean silique size harvested from each non-induced and induced plant; and (**c**) Mean number of siliques harvested from non-induced and induced plants. Wt: wild-type *A. thaliana*; Ye: *A. thaliana* (*yefM_Spn_*); Yg: *A. thaliana* (*yoeB_Spn_-GFP*); Hy: *A. thaliana* (*yefM_Spn_* × *yoeB_Spn_-GFP*) hybrid. Data in (**b**,**c**) are presented as the mean ± standard deviation (*n* = 20); significance values (with significant differences between samples indicated by the letters a, b and c) were determined by one-way ANOVA (Tukey used as the *post hoc* test with *p* < 0.05).

Before induction, the growth of the *yefM_Spn_* transgenic plants, *yoeB_Spn_-GFP* transgenic plants and *yefM_Spn_* × *yoeB_Spn_-GFP* hybrid plants was similar to that of the untransformed control plants (Figure 6a). By the seventh day post-induction, transgenic plants expressing the *yoeB_Spn_-GFP* fusion had died (Figure 6 and Figure 7; as we had reported previously in [16]). However, hybrid transgenic plants co-expressing both the *yoeB_Spn_*-*GFP* fusion and the *yefM_Spn_* antitoxin gene remained healthy, indicating that co-expression of the *yefM_Spn_* antitoxin was able to neutralize the lethality of the *yoeB_Spn_* toxin (Figure 6a). Transgenic plants expressing *yoeB*_Spn_ showed characteristic DNA fragmentation patterns indicative of apoptosis [16]. The DNA fragmentation assay showed that no fragmentation was observed in the 17-β-estradiol-induced transgenic hybrid plants (Figure S1). Interestingly, in all three independent hybrid lines, the plants induced to express both *yefM_Spn_* and *yoeB_Spn_-GFP* displayed increased growth in terms of height, number of branches and inflorescence stems (Figure 6c–e), as well as rosette leaf size at the full stage of maturity, five weeks after 17-β-estradiol induction (*i.e.*, nine weeks post-planting). The growth of each rosette leaf in the hybrid plants exceeded that of the leaves from the non-induced and induced control plants (*i.e.*, *yefM_Spn_*, *yoeB_Spn_-GFP* and wild-type plants); both the petiole length and width of the rosette leaves were greater and significantly increased in the induced hybrid plants (Figure 6b snd Figure S2). The increased growth of the hybrid *A. thaliana* plants was also reflected in the significant increase in the dry weight (Figure 6f).

At nine weeks post-planting (*i.e.*, five weeks after induction), the differences in the length of siliques were, however, not significant (Figure 7a,b). Nevertheless, the number of siliques per induced hybrid plants was significantly higher (up to 50%) than that of all control plants (Figure 7c), except for *the yoeB_Spn_-GFP* transgenic plants that had died after the first week of induction, and therefore, no measurement could be recorded.

## 3. Discussion

The *yefM-yoeB_Spn_* TA system from the bacterial pathogen *S. pneumoniae* has been characterized and shown to be functional in its native host, as well as in *E. coli*, where over-expression of the *yoeB_Spn_* toxin, an endoribonuclease, was found to be inhibitory to cellular growth [12,15,18]. In our earlier study, we showed that the *yoeB_Spn_* toxin-*GFP* fusion was functional and toxic in *Arabidopsis thaliana* plants, as its induced expression was associated with signs of apoptosis [16]. The current study aimed to determine whether co-expression of the *S. pneumoniae yefM*_Spn_ antitoxin with the *yoeB_Spn_* toxin-*GFP* fusion in *A. thaliana* could neutralize the lethal effects of the toxin. While over-expression of *yefM_Spn_* in *E. coli* reportedly inhibited growth [15], we found that the induced expression of the *yefM_Spn_* antitoxin alone in *A. thaliana* did not adversely affect the plants, nor were there any morphological differences between the wild-type, transgenic induced and non-induced plants up to five weeks after induction (Figure 3 and Figure 6). The lack of any change in transgenic *A. thaliana* expressing *yefM_Spn_* is an important observation, as when expressed together with the toxin, clear changes in phenotype were evident (Figure 6).

In this study, we performed sexual crosses to obtain hybrid plants containing both *yoeB_Spn_* toxin-*GFP* and *yefM_Spn_* antitoxin constructs. Co-expression of the *yefM_Spn_* and *yoeB_Spn_-GFP* enabled hybrid plants to thrive (Figure 6), in contrast to expression of *yoeB_Spn_* toxin-*GFP* alone, which was lethal [16]. This indicated that the *yefM_Spn_* antitoxin was able to neutralize the *yoeB_Spn_* toxin-*GFP* fusion in *A. thaliana*. A study carried out by Nieto *et al.* [15] has shown that the lethal action of the pneumococcal YoeB_Spn_ toxin was neutralized by tight binding with its cognate YefM_Spn_ antitoxin in its native host cell, as well as *E. coli*; we suggest that YefM_Spn_ and YoeB_Spn_-GFP in *A. thaliana* behaved similarly. Our previous work revealed that the *yoeB_Spn_* toxin-*GFP* fusion mRNA was expressed in transgenic *A. thaliana*, peaking at three days after induction, after which it decreased [16]. In this study, induction of either of the *yoeB_Spn_* toxin-*GFP* fusion or *yefM_Spn_* antitoxin constructs in separate hybrid plants showed similar stable expression with the *yefM_Spn_* transcript levels also peaking three days after induction. The *yoeB_Spn_-GFP* expression levels were also relatively lower than that of its cognate antitoxin, *yefM_Spn_* (Figure 5b), in the hybrid plants, the reason for which is currently unknown.

Interestingly, the induced expression of *yefM_Spn_* and *yoeB_Spn_-GFP* constructs when together in hybrid plants led to unexpected phenotypic effects in the growth and morphology of *A. thaliana*. The major alterations were seen in larger rosette leaves, taller plants with higher number of inflorescence stems and increased silique production, as compared to the wild-type, transgenic induced and non-induced *yefM*_Spn_, transgenic induced and non-induced *yoeB_Spn_-GFP* and to the non-induced hybrid control plants. It is likely that the larger rosette leaves provide more of the photosynthate needed for a higher number of inflorescence stems and seed development, thereby leading to an increase in silique production. The reasons and mechanisms are not known, but some of the possible pathways that could be affected are plant hormones, water-use efficiency, mineral uptake and photosynthetic efficiency. In *S. pneumoniae*, the YefM_Spn_ antitoxin also functions as a transcriptional autorepressor by binding to a palindrome sequence that overlaps the promoter for the *yefM-yoeB*_Spn_ operon. The YoeB_Spn_ toxin functions as a co-repressor by enhancing the binding of YefM_Spn_ to its operator site when it is in a YefM-YoeB_Spn_ protein complex [18]. Thus, the YefM-YoeB_Spn_ protein complex has DNA-binding capabilities, and it is thus possible that binding of the YefM-YoeB-GFP protein complex to certain sections of the Arabidopsis genome in the transgenic hybrids could have led to the enhanced growth phenotype, as indicated in Figure 6 and Figure 7. To explore the possibility that the *A. thaliana* genome contained similar sequences to the native YefM-YoeB_Spn_ binding sites, the 27-nucleotide binding sequence of the protein complex obtained through DNase I footprinting assays [18] was used as the query in a BLASTN search of the *A. thaliana* genome sequence [19]. We found 10 *A. thaliana* loci that had 18–21 nucleotide matches to the 27-nucleotide YefM_Spn_-binding motif (Table S1). None appeared to be within gene promoter or enhancer regions. The highest nucleotide identities (at 21 out of 27 nucleotides) were found within the *ARPC5* gene (GI: 240256243), which codes for an actin-related protein 2/3 complex, subunit 5A, and that plays a role in cell morphogenesis, plant growth and development [20]. Two of the matching sequences (at identities of 19 out of 27 nucleotides) were found to belong to genes coding multidrug and toxic compound extrusion (MATE) efflux family proteins (GIs: 240256493 and 240254678) that have been reported to modulate genes involved in plant growth and development, as well as conferring defense mechanism against biotic stress [21]. As far as can be ascertained, neither the YefM_Spn_ nor the YoeB_Spn_-GFP proteins (with estimated molecular weights of 9.7 and 37.6 kDa, respectively, as determined using ProtParam) contained any recognizable nuclear localization sequence (NLS), and the estimated size of the putative complex is within the limit of size to allow for nuclear transport (*i.e.*, 90–110 kDa; [22]). Nevertheless, future studies using structural molecular models for protein-DNA binding of the putative complex to the Arabidopsis genome might shed more light on the mechanism, as using the DNA sequence alone has a limited ability for the confirmation of suitable binding sites.

The detailed mechanism by which co-expression of *yoeB_Spn_-GFP* and *yefM_Spn_* led to enhanced plant growth remains to be elucidated and is a subject for further research. This study has demonstrated that co-expressing the pneumococcal *yoeB_Spn_* toxin gene with its cognate *yefM*_Spn_ antitoxin gene was able to neutralize the lethality of the YoeB_Spn_ toxin in transgenic *A. thaliana*. To our knowledge, this is the first demonstration of a prokaryotic antitoxin neutralizing its cognate toxin in plant cells. In addition, the enhanced growth phenotype of the transgenic hybrid plants co-expressing the YefM_Spn_ and YoeB_Spn_ proteins is an attractive motivation to pursue research along this line for potential biotechnological applications.

## 4. Materials and Methods

### 4.1. Construction of Plasmids

This study used the plant inducible responder vector pMDC150_35S, which contains the CaMV 35S promoter [16], and activator vector pMDC160_*yefM_Spn_*, which contains the *yefM_Spn_* antitoxin gene. To develop pMDC160_*yefM_Spn_*, the *yefM_Spn_* antitoxin coding sequence from *S. pneumoniae* was amplified by PCR from the construct pET28a_HisYefMYoeB [18] with primers yefM_F: 5′-CACCATGGAAGCAGTCCTT-3′ and yefM_R: 5′-TCACTCCTCAATCACATGGA-3′. The PCR-amplified *yefM_Spn_* was inserted into the Invitrogen Gateway^®^ pENTR_D_TOPO cloning vector (Invitrogen, Thermo Fisher Scientific, Waltham, MA, USA) according to the manufacturer’s instructions and transformed into *E. coli* TOP10-competent cells. The presence of the insert was confirmed by colony PCR using the primers M13_F: 5′-GTAAAACGACGGCCAG-3′ and yefM_R; which resulted in an amplicon of approximately 455 bp. The plasmids were extracted from positive colonies and verified by conventional Sanger DNA sequencing prior to cloning the *yefM_Spn_* antitoxin coding sequence into the Gateway^®^ pMDC160 destination vector [17] via LR Clonase using Gateway^®^ technology. The two expression constructs, pMDC150_35S and pMDC160_*yefM_Spn_*, were separately transformed into *Agrobacterium tumefaciens* strain LBA4404 using a freeze and thaw method [23]. Antibiotic resistance was used to select the transformed colonies.

### 4.2. Plant Material and Growth Condition

*Arabidopsis thaliana* ecotype Columbia 0 was used in all experiments. Seeds were stratified for 3 days at 4 °C and sown on soil. Plants were grown in a growth room with a 16-h photoperiod under 70% relative humidity at 22 °C. The two control plants used in this study were wild-type and non-induced transgenic Arabidopsis (*yoeB_Spn_-GFP*, *yefM_Spn_* and *yefM_Spn_* × *yoeB_Spn_-GFP* hybrid).

### 4.3. Plant Transformation and Selection

*Agrobacterium tumefaciens*-mediated transformation of *A. thaliana* with both recombinant constructs pMDC150_35S and pMDC160_*yefM_Spn_* was performed using a double floral dip method as described by [24]. A total of 5 independent transformation events were conducted, from which 3 transgenic lines were used for further analysis. Seeds were harvested and grown under antibiotic and/or herbicide selection until the T_2_ generation. For selection of transgenic Arabidopsis plants that are resistant to the antibiotic kanamycin (for pMDC150_35S) and the herbicide Basta (glufosinate) (for pMDC160_*yefM_Spn_*), seeds were stratified for 3 days at 4 °C before sowing on soil. The germinated seeds were grown for 1 week before spraying with a mixture of 50 mg/L kanamycin and 0.25 mg/L Basta. The antibiotic-herbicide mixture was applied at 3-day intervals for 2 weeks. Surviving seedlings with resistance to kanamycin and Basta were transferred to new soil until maturity. For selection of transgenic Arabidopsis that were resistant to kanamycin (for pMDC150_35S) and hygromycin (for pMDC221_*yoeBGFP*), the concentration used was 50 mg/L for each antibiotic and applied using the same regimen as above.

### 4.4. PCR Analysis

Genomic DNA was isolated from the rosette leaves using a cetyl trimethylammonium bromide (CTAB) method, as previously described [25]. PCR analysis was performed to confirm the presence of the entire gene cassette in transgenic plants using primers Transg_yefM_F: 5′-ATGGAAGCAGTCCTTTACTCA-3′ with Transg_yefM_R: 5′-TCACTCCTCAATCACATGGA-3′ for the *yefM_Spn_*-expressing plants and Transg_yoeB_F: 5′-CACCATGCTACTCAAGTTTA-3′ with Transg_GFP_R: 5′-TTATAATCCCAGCAGCTGTT-3′ to detect *yoeB_Spn_-GFP* transgene for the *yefM_Spn_* × *yoeB_Spn_-GFP* hybrid plants. The same Transg_yefM primers were also used to detect the presence of *yefM_Spn_* transgene in the hybrid plants. PCR reaction mixtures consisted of 50 ng genomic DNA, 0.5 µM of each primer, 1× GoTaq^®^ Green Master Mix (Promega Corporation, Madison, WI, USA) and MilliQ water to make up the total volume of 25 µL. The amplification protocol was as follows: initial denaturation at 95 °C for 2 min, 32 cycles of incubation at 95 °C for 30 s, 48 °C for 30 s, 72 °C for 1 min and one final extension at 72 °C for 5 min.

### 4.5. Morphology of Transgenic A. thaliana

Four-week old transgenic *A. thaliana* transformed with pMDC150_35S and pMDC160_*yefM_Spn_* were induced using 100 μM 17-β-estradiol as described by [16]. The induced, non-induced and wild-type plants were observed each day from Day 1 after induction until Day 9. A total of 68 T_2_ transgenic plants from 3 different T_1_ lines were used in this study. For the transgenic hybrid *A. thaliana* harboring *yefM_Spn_* and *yoeB_Spn_-GFP*, the same amount of 17-β-estradiol was applied, and the plants were allowed to grow until reaching full maturity (where all siliques were fully formed) before recording phenotypic measurements. Phenotypic measurements of induced, non-induced and wild-type plants (*n* = 20 for each group) included length of rosette leaves, height (measured as the length from the soil to the top of each plant), number of inflorescence stems formed in each plant, number of branches bearing siliques, measurement of dry weight, length of siliques and total number of siliques harvested per plant.

### 4.6. Statistical Analysis

Data were analyzed using ANOVA with SPSS for Windows, Version 16.0. (SPSS Inc., Chicago, IL, USA). A significant difference from the control value(s) was determined at *p* < 0.05 levels. All reported data represent the mean ± SD of at least three independent experiments.

### 4.7. Cross-Pollination and Selection of Hybrid T_2_ Seeds

The flowers of transgenic *A. thaliana* plants from T_1_ generations harboring pMDC150_35S/pMDC160_*yefM_Spn_* and pMDC150_35S/pMDC221_*yoeB_Spn_-GFP* were used as the ovule donor and pollen donor, respectively. Unopened flower buds were sliced open lengthwise and emasculated with sterilized forceps. Mature pollen from the donor plant was transferred onto the stigmas of emasculated plants by brush. The cross-pollinated flowers were marked and wrapped with small plastic bags to prevent additional pollination from other pollen sources. The plants were grown under the same conditions as described above until the seeds were ready to be collected. The seeds were stratified and selected on hygromycin, kanamycin and Basta, as described above. The surviving seedlings were then transplanted and grown under non-selective conditions until they reached four weeks old before further analysis. Eleven lines of hybrid plants were produced from the cross-pollination.

### 4.8. Southern Blot Analysis

Total genomic DNA (20 μg) from both wild-type and hybrid *A. thaliana* was digested with *Eco*RI for 48 h and separated in 0.7% (*w*/*v*) agarose gel. Digested DNA was transferred to a small strip of a positively-charged nylon membrane (Roche Diagnostics, Indianapolis, IN, USA) and hybridized with probes derived from the *yoeB_Spn_-GFP* (732 bp) and *yefM_Spn_* (255 bp) PCR products. All blotting procedures and immunological detection were carried out according to the DIG DNA Labeling and Detection Kit application manual (Roche Diagnostics).

### 4.9. qRT-PCR Analyses of yefM_Spn_ and yoeB_Spn_-GFP Expression in Transgenic Hybrid Plants

Total RNA was extracted from Arabidopsis rosette leaves each day from Days 1–7 after induction with 100 μM 17-β-estradiol (as described above) using RNeasy Plant Mini Kit (Qiagen, Düsseldorf, Germany) and according to the manufacturer’s protocol. To remove traces of DNA contamination, 1 μg of the isolated RNA was treated with DNase 1 using QuantiTect^®^ Reverse Transcription Kit (Qiagen, Germany). cDNA was then synthesized from 1 μg of the treated RNA in two steps using a QuantiTect^®^ Reverse Transcription Kit (Qiagen, Germany) under the following conditions: 42 °C for 15 min followed by inactivation at 95 °C for 3 min. Master Mix (20 µL) was prepared according to the manufacturer’s protocol. qRT-PCR was performed in a final volume of 20 µL, which consisted of 0.5 µM of both forward and reverse primers each, 25 ng of cDNA as the template and 1× SYBR Green Master mix (Applied Biosystem, Foster City, CA, USA) using a QuantStudio™ 12K Flex Real-Time PCR System (Qiagen, Düsseldorf, Germany). After PCR, the data were quantified using the comparative *C*_t_ method (2^∆∆*C*t^) [26]. The expression of *yefM_Spn_* was determined using the primers q-yefM-F: 5′-AGCCTTTGACGGTGGTCAATAA-3′ and q-yefM-R: 5′-AGCACGGACTTGAGCCATTC-3′; whereas the expression of *yoeB_Spn_-GFP* was measured using the primers q-yoeB-F: 5′-GGACGACGGGAACTACAAGA-3′ and q-yoeB-R: 5′-CGGCCATGATGTATACGTTG-3′. The expression level from the Day 1 sample was used as the calibrator (value of 1.0). Each gene was assayed using three biological replicates. The A. thaliana actin gene was amplified using the q-Actin-F primer: 5′-CCAGTGGTCGTACAACCGGTAT-3′ and q-Actin-R primer: 5′-ACCCTCGTAGATTGGCACAGT-3′ and was used as the reference to normalize gene expression across the samples.

## Supplementary Materials

Supplementary materials can be found at http://www.mdpi.com/1422-0067/17/4/321/s1.

## Abbreviations

CTAB: Cetyl trimethylammonium bromide
GFP: Green fluorescent protein
qRT-PCR: Quantitative real-time reverse transcriptase PCR
TA: toxin-antitoxin
