# Supplementary material for: Neutralization of Bacterial YoeBSpn Toxicity and Enhanced Plant Growth in Arabidopsis thaliana via Co-Expression of the Toxin-Antitoxin Genes
Source: Int J Mol Sci. 2016 Apr 20;17(4):321. doi: 10.3390/ijms17040321 (PMC4848878; doi:10.3390/ijms17040321)
Supplement: Supplementary file 1 [file ijms-17-00321-s001.pdf]

Supplementary Material: Neutralization of YoeB$_{Spn}$ Toxicity and Enhanced Plant Growth in *Arabidopsis thaliana* via Co-Expression of Toxin-Antitoxin Genes

Fauziah Abu Bakar, Chew Chieng Yeo and Jennifer Ann Harikrishna

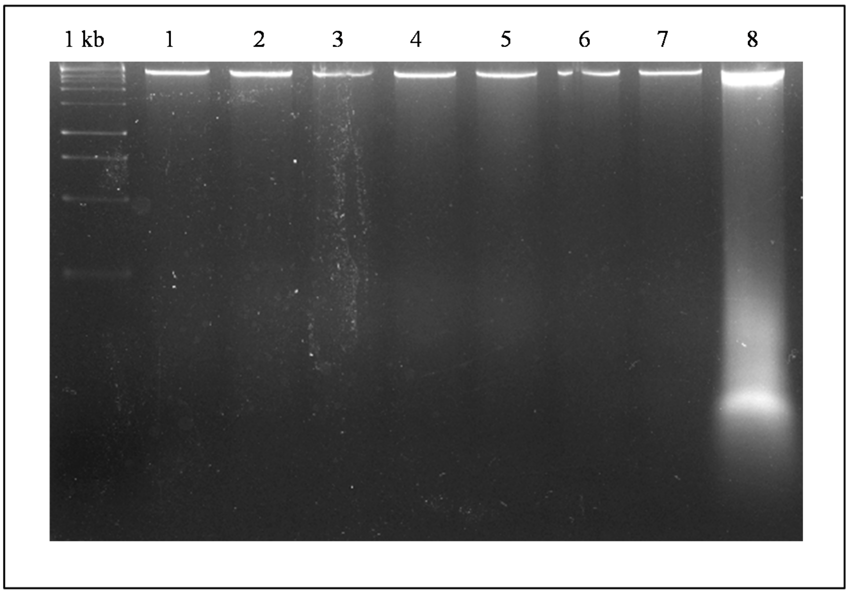

Figure S1. Laddering of nuclear DNA from transgenic *yoeB$_{Spn}$-GFP*. Lane **1**: Non-induced wild-type; **2**: Non-induced *yefM$_{Spn}$*; **3**: Non-induced *yefM$_{Spn}$* × *yoeB$_{Spn}$-GFP* hybrid; **4**: Non-induced *yoeB$_{Spn}$-GFP*; **5**: Induced wild-type; **6**: Induced *yefM$_{Spn}$*; **7**: Induced *yefM$_{Spn}$* × *yoeB$_{Spn}$-GFP* hybrid; **8**: Induced *yoeB$_{Spn}$-GFP*; 1 kb: 1 kb DNA ladder (Fermentas).

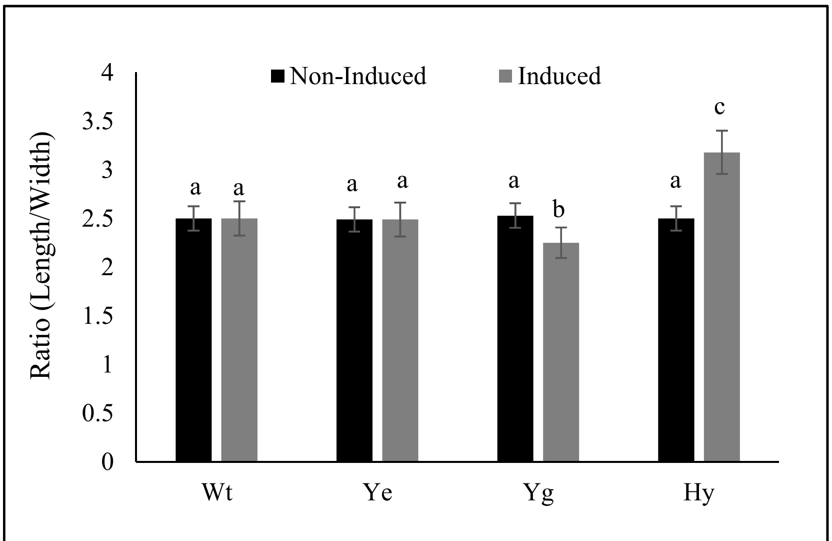

Figure S2. Ratio of length to width of leaf (7th position) of induced and non-induced plants. Wt: wild-type *A. thaliana*; Ye: transgenic *A. thaliana* (*yefM$_{Spn}$*); Yg: transgenic *A. thaliana* (*yoeB$_{Spn}$-GFP*); Hy: transgenic *A. thaliana* (*yefM$_{Spn}$* × *yoeB$_{Spn}$-GFP*) hybrid. Data are presented as the mean ± standard deviation ($n = 20$); significance values (with significant differences between samples indicated by the letters a, b and c) were determined by one-way ANOVA (Tukey used as the *post hoc* test with $p < 0.05$).

Table S1. Possible binding sites for the YefM-YoeB$_{Spn}$-GFP complex in the *Arabidopsis thaliana* genome based on the 27-nucleotides binding site for YefM-YoeB$_{Spn}$ in *S. pneumoniae* as determined by DNaseI footprinting by Chan *et al.* [18].

Accession No.	Chromosome No.	Identities (Out of 27 Nucleotides)	Gene	Features	Possible Molecular Function(s)
GI/240256243 ref/NC_003075.7	4	21	*ARPC5*	Actin related protein 2/3 complex, subunit 5A	Regulation of actin polymerization and together with an activating nucleation—promoter factor (NPF)
GI/240255695 ref/NC_003074.8	3	20	Representative gene model—At3g31051.1	Hypothetical protein	Involved in transcription or purifying selection
GI/2402544121 ref/NC_003070.9	1	20	*UBP13*	Ubiquitin carboxyl terminal hydrolase related protein	Cystein type peptidase activity; Ubiquitin—dependent protein catabolic process
GI/240256493 ref/NC_003076.8	5	19	1. MATE (Multidrug and toxic compound extrusion)<br>2. Representative gene model—At2g16870	1. MATE efflux family protein<br>2. TIR-NBS-LRR class disease resistance protein	1. Citrate exudation into the rhizosphere, drug transmembrane transporter activity.<br>2. ADP/ATP binding
GI/240256243 ref/NC_003075.7	4	19	Representative gene model—At4g37095.1	Hypothetical protein	Gene codes for small protein either involved in transcription or purifying selection
GI/240255695 ref/NC_003074.8	3	19	*T21J18-100*	Polynucleotide adenyltransferase domain and RNA recognition motif containing protein	Nucleotide binding nucleotidyltransferase activity
GI/240254678 ref/NC_003071.7	2	19	TT12/DTX14 Representative gene model—At3g59030	MATE efflux family protein (F3L12.8)	Citrate exudation into the rhizosphere, drug transmembrane transporter activity
GI/240254678 ref/NC_003071.7	2	19	*TT12/DTX14* Representative gene model—At3g59030	MATE efflux family protein	Citrate exudation into the rhizosphere, drug transmembrane transporter activity
GI/240254421 ref/NC_003070.9	1	19	1.Representative gene model—At1g34930<br>2. *LAX3*	1. Plant thionin family protein<br>2. Auxin influx carrier LAX3	1. Cystein rich protein with toxic and antimicrobial properties involved in plant defense mechanism.<br>2. Promotes lateral root emergence and is involved in phlotaxis arrangement of leaves and stem
GI/240254678 ref/NC_003071.7	2	18	*APC2* Representative gene model—At2g04660.1	Anaphase promoting complex subunit—2	A highly conserved ubiquitin protein ligase involved in cell cycle regulation and also promotes protein binding
